# Quantifiable effects of regular exercise on zinc status in a healthy population—A systematic review

**DOI:** 10.1371/journal.pone.0184827

**Published:** 2017-09-20

**Authors:** Anna Chu, Trishala Varma, Peter Petocz, Samir Samman

**Affiliations:** 1 Department of Human Nutrition, University of Otago, Dunedin, New Zealand; 2 Department of Statistics, Macquarie University, Sydney, New South Wales, Australia; 3 School of Life and Environmental Sciences, University of Sydney, Sydney, New South Wales, Australia; TNO, NETHERLANDS

## Abstract

Zinc is an essential mineral of which its functions have potential implications on exercise performance and beneficial adaptations of physical activity. While the effects of aerobic exercise on zinc metabolism acutely have been well described, the effect of long-term exercise training on zinc status remains unclear. The present review aims to determine the effects of exercise training on markers of zinc status in an apparently healthy adult population. We conducted a systematic literature search on PubMed, Scopus, SPORTDiscus and Cochrane Library from inception to 28 January 2016 to identify interventional or cohort studies that investigated the effects of exercise training on indices of zinc status. Pairwise comparisons of mean differences in within-group change were calculated and summarised visually in forest plots. Six studies satisfied the inclusion criteria for the systematic review, of which 5 studies included data on changes in serum zinc concentrations and 3 studies provided changes in dietary zinc intake. Two comparisons showed significantly higher increase of serum zinc concentrations in the exercise group compared to control, while one comparison reported significantly lower change in serum zinc for the exercising group. The exercise groups consumed significantly higher dietary zinc compared to controls in two comparisons. The present review revealed an incomplete evidence base in evaluating the effect of long-term exercise training on markers of zinc status. Further well-designed investigations are required to elucidate the relationship for establishment of dietary recommendation in populations who are continuing exercise interventions.

## Introduction

Zinc is an essential trace element with numerous metabolic functions [1]. While zinc is found ubiquitously in the human body, a significant portion of zinc is located within the musculoskeletal system [2]. In the context of exercise, zinc provides structural integrity and supports catalytic functions for metalloenzymes, such as carbonic anhydrase, superoxide dismutase (SOD) and lactate dehydrogenase [3]. Furthermore, zinc regulates intracellular signalling pathways and corresponding downstream effects on immune function [4,5] and redox homeostasis [6,7], with potential implications for performance [8] and related metabolic benefits of exercise [9].

Exercise and physical activity have been the cornerstone of lifestyle recommendations for the healthy population and those with chronic diseases [10,11]. The public health recommendations for physical activity are based on the benefits of exercise on metabolic, musculoskeletal and neuromotor health [12]. Metabolic adaptations of exercise can be modulated by nutritional status, such as the availability of macronutrients, specifically protein and carbohydrates [13]. The role of adequate micronutrient status in supporting the beneficial adaptations of exercise has gained research attention [14], particularly with respect to the effects of physical activity on the zinc status and subsequent consequences on exercise performance and metabolic effects [15].

A number of cross-sectional studies have investigated the association between exercise/physical activity and zinc status, in particular the zinc status in athletic and control populations. While some studies showed lower serum zinc concentration in athletes [16,17], other papers report no significant differences in zinc status between athletes and controls [18,19]. The lack of conformity in the results may be driven by factors other than physical activity levels, for example differences in dietary habits between the populations. The current evidence is inconclusive in determining the relationship between exercise and zinc status in cross-sectional data, however the examination of longitudinal and cohort studies may be able to elucidate the potential relationship.

We have previously reported significant acute fluctuations in serum zinc concentrations as a result of a single bout of aerobic exercise [20,21]. The changes in zinc metabolism were proposed to be influenced by the events that occur during exercise and recovery, including leakage of zinc ions from damaged myocytes and exercise-induced inflammatory processes that follow aerobic exercise. Preliminary evidence suggests that training status is a modulating factor for the acute effects of exercise on zinc metabolism [20], but it is currently unclear whether exercise training itself imposes adaptations of zinc status and/or metabolism during exercise. Aerobic exercise has been shown to significantly increase systemic zinc concentration immediately following the exercise bout with decline of zinc concentration to below the baseline values in the hours after aerobic exercise; it is uncertain whether the acute exercise-induced changes in zinc metabolism are sustained in the long term. Therefore, the current review aims to determine the long term effects of exercise training on markers of zinc status in an apparently healthy adult population, as identified by interventional or cohort trials.

## Methods

### Search strategy

**Fig 1:**
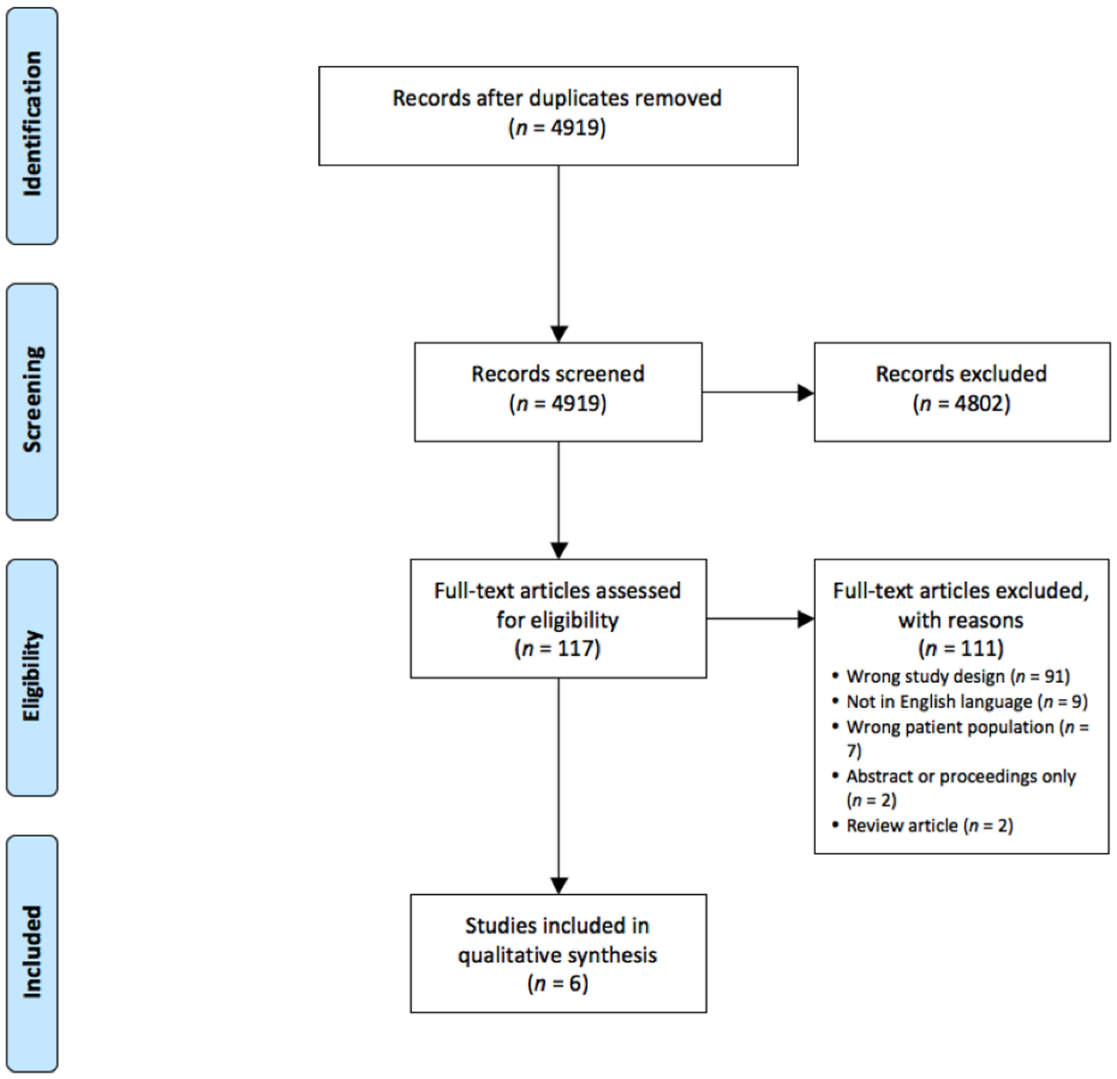
PRISMA diagram showing the systematic review process.

A literature search of PubMed, Scopus, SPORTDiscus and the Cochrane Library electronic databases were conducted from inception to the 28^th^ of January 2016. The search terms included: zinc, exercis*, athlet*, physical activity, train*. Related terms and MeSH terms were used where appropriate. Searches were limited to human subjects and the English language. Fig 1 shows the Preferred Reporting Items for Systematic reviews and Meta-Analyses (PRISMA) flowchart describing the electronic search outcomes and selection process [22]. Review questions, search strategies and inclusion criteria for this review were prospectively specified and registered with PROSPERO at http://www.crd.york.ac.uk/PROSPERO/ (CRD42015026336).

#### Study inclusion criteria

Interventional and cohort studies that were published in peer-reviewed journals and examined the effects of exercise training on zinc status were included. Exercise training was defined as an intervention, over a defined period of time, which involves more than one bout of continuous physical activity [23]. Males and females, aged between 18 and 65 years, who were apparently healthy and not diagnosed with any major health condition or illness were included in the present review. The included studies must include a control group and also report indices of zinc status, such as dietary zinc intake, zinc-related enzymes and/or concentrations of zinc measured in serum/plasma, urine, erythrocytes, hair and nail, before and after the exercise training period. Two investigators independently reviewed each citation, and where appropriate, each full report, to determine whether the study satisfy the inclusion criteria.

### Data extraction and quality assessment of included studies

Two investigators extracted published data from all included studies independently, and any differences were resolved by discussion. Data extracted included descriptive information, such as the study authors, year of publication, country where the study was conducted, the number, sex and other characteristics of the control and study populations. The duration, intensity and mode of exercise performed in the study were detailed in the data extraction process. Primary outcomes extracted were plasma/serum zinc concentration and dietary zinc intake, before and following exercise training. Plasma and serum concentrations were grouped to represent systemic zinc concentration; in this report the term “serum” will be used to represent both serum and/or plasma. Other potential markers of zinc status, including zinc concentrations in urine and erythrocyte, and activity or concentration of Cu-Zn superoxide dismutase were extracted as secondary outcomes.

### Risk of bias assessment

All included studies were assessed for risk of biases that are applicable for non-randomised interventional studies as recommended by the Grading of Recommendations Assessment, Development and Evaluation (GRADE) guidelines [24]. Two independent reviewers assessed the potential risk of biases including recruitment bias, valid measurement of primary outcomes (serum zinc concentration and dietary zinc intake), incomplete accounting for participants in control or exercising groups, and selective outcome reporting. The ratings of risk included *low*, *unclear* and *high*. Studies with exercise group matched with control group in age, sex and body composition represent a *low* risk of bias in recruitment. Valid measurement of serum zinc was determined by the use of trace element free collection tubes, appropriate method of analysis i.e. atomic absorption spectroscopy or inductively coupled plasma mass spectroscopy, and timing of blood collection. Similarly, *low* risk of bias for the valid measurement of dietary zinc was achieved by the use of appropriate method of collection (food record or 24 h recall), reporting of energy or macronutrient intake and consideration of supplemental intake. Data on risk of bias in individual studies were entered into Review Manager 5.3 [25].

### Statistical analyses

Changes in the primary outcome measures of serum zinc concentration and dietary zinc intake were calculated by subtracting final from baseline values, with calculated estimation of SD of change using an assumed correlation of 0.7 between the two values [26]. The differences between exercise and control groups were calculated by subtracting pre-post change in the exercise group from pre-post change in the control group. Standard error of between-group differences was estimated from calculation using SD of change and number of participants in the two groups. Pairwise comparisons of mean differences in change between the exercise and control groups were calculated in Review Manager 5.3 [25] and summarised visually in forest plots. Meta-analysis for the effect of exercise training on zinc status was deemed inappropriate due to the variations in the exercise training reported.

## Results

The electronic database search identified a total of 4919 citations following removal of duplicates. After initial screening of titles and abstracts, 4802 citations were excluded as they were irrelevant to the current review. Of the remaining 117 full texts assessed for eligibility, six studies met the inclusion criteria. Fig 1 shows the details of study selection and reasons for full text exclusion.

### Study characteristics

The characteristics of studies included in the current review are described in Table 1. Three of the included studies were prescribed exercise interventions [27–29] whereby two studies assigned specific exercise interventions to sedentary [28] or athletic populations [29]; another study randomised untrained men into either an exercise group or a control group [27]. The remaining three cohort studies examined athletes before and after a period of time that was relevant for their sport, i.e. competition season [30,31] and a 20 day sailing race [32]. The types of exercise in the included studies were heterogeneous; five of the included studies included exercise that is mostly in aerobic activity [28–32] while one study included a resistance exercise program [27]. The duration of the exercise training was between 20 days and 6 months. The number of total participants in each study ranged from 20 to 75. The majority of the studies examined males [27,29,30,32], with the exception of two studies [28,31]. The relevant zinc outcomes reported in the included studies comprise of zinc concentration in serum [28,30–32], urine [30] and erythrocyte [28], dietary zinc intake [27,30,31], and erythrocyte Cu,Zn- SOD [27,31]. Other zinc-dependent enzymes, such as lactate dehydrogenase or carbonic anhydrase, were not reported in the included studies.

**Table 1:** Characteristics of included studies.

Study (author, year)	Study type^1^	Study group	Control group	Study *n*	Study Age (y)^2^	Control *n*	Control Age (y)	Sex (M/F)	Exercise training	Indices of zinc status
Azizbeigi *et al*. 2013	I, R	Untrained men	Untrained men	10	21.1 ± 2.1	10	23.3 ± 2.5	M	Progressive resistance exercise training 3 x/week for 8 weeks	Dietary, SOD^3^
Cordova & Navas 1998	C	Spanish League of Volleyball players	Moderately trained university students	12	25.9 ± 2.6	12	22.3 ± 1.2	M	Volleyball season training, 5 h/d, 7 x/week, approximately 8 weeks	Dietary, serum, urine
Fogelholm *et al*. 1991	C	Sailors	Bank clerks	14	28 ± 0.27	11	33 ± 0.6	M	Transatlantic sailing for 20 d	Serum
Fogelholm 1992	I, NR	University students	University students	21	24 ± 0.6	18	26 ± 0.6	F	Progressive aerobic exercise training for 24 weeks, from 2 x/week to 6 x/week, 30–45 min/d at 60–80% HRR	Serum, erythrocyte
Lukaski *et al*. 1990	C	Varsity swimmers	Non-training university students	13	NS	15	NS	M	Competitive swimming season for 24 weeks, not specified	Dietary, plasma, SOD
		Varsity swimmers	Non-training university students	16	NS	13	NS	F	Competitive swimming season for 24 weeks, not specified	Dietary, plasma, SOD
Peake *et al*. 2003	I, NR	Well-trained distance runners	Sedentary males	10	28 ± 7	7	21 ± 0	M	16% increase in running training volume over 4 weeks	Plasma

^1^ C, cohort; I, interventional; NR, not randomised; R, randomised;

^2^ presented as mean ± SD; NS, not specified;

^3^ SOD, superoxide dismutase

### Change in serum zinc concentration

**Fig 2:**
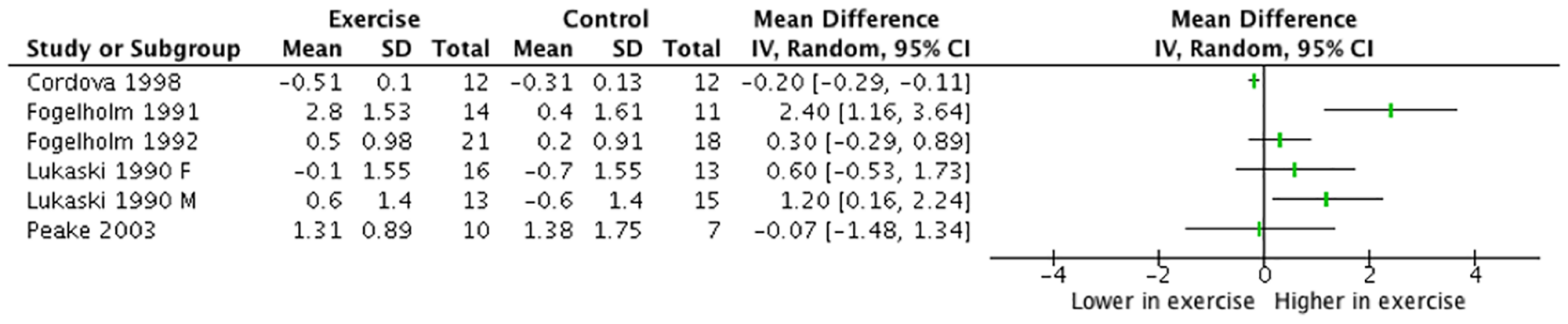
Pairwise comparisons of the change in serum zinc concentration (μmol/L) between exercise and control groups in interventional trials. Data are presented as mean difference (95% CI).

Five studies [28–32] provided data on changes in serum zinc concentrations for six comparisons. Statistical significance of between-group changes in serum zinc concentration were not reported for three studies [29–31], while one study reported non-significant increases in serum zinc concentration in both exercise and control groups [28]. One study found a significantly higher change in serum zinc concentration in the exercising group compared to the control group [32]. Fig 2 shows a forest plot that summaries the mean difference ± 95% CI in serum zinc concentration between the exercise and control groups. The mean differences of change ranged from -0.20 to 2.40 μmol/L; two out of six comparisons elicited significantly higher increase of serum zinc concentrations in the exercise group compared to control [31,32], while one comparison reported significantly lower change in serum zinc for the exercising group [30]. Two studies reported serum zinc concentration with comparison to the reference range. No participants presented with plasma zinc concentration below the reference range in Lukaski *et al*.’s study. A small percentage of participants displayed low serum zinc concentration in another study [28], with 5% of the exercise group presenting with low serum zinc at all time points and 6% of the control group only at the end of the intervention period.

### Change in dietary zinc intake

**Fig 3:**
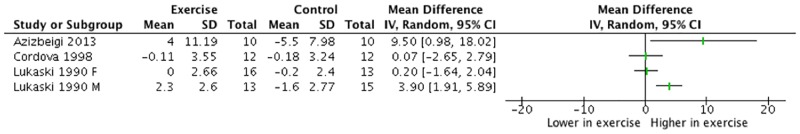
Pairwise comparisons of the change in dietary zinc intake (mg/d) between exercise and control groups in interventional trials. Data are presented as mean difference (95% CI).

Three studies [27,30,31] provided data on change in dietary zinc intake for four comparisons. The results from the included studies did not include statistical differences of the change in dietary zinc intake between the exercise and control group. Forest plot summarising the calculated between group mean difference ± 95% CI of dietary zinc intake is shown in Fig 3. The mean differences of change ranged from 0.07 to 9.5 mg/d; the exercising groups consumed significantly higher dietary zinc compared to controls in two out of four comparisons. Two studies reported dietary zinc intake of participants with comparisons to the recommended intakes. Lukaski *et al*. [31] reported dietary zinc intake of control females only to be lower than 67% of RDI at both preseason and postseason. Fogelholm [28] reported that 21% of those in the exercising group were consuming dietary zinc intakes below the Nordic recommendations during the intervention; similarly, 27% of the control group presented with low dietary zinc intake. In the only study that reported zinc density [31], no significant changes were noted in zinc density within the diets before and after the competitive swimming season in both male and female participants.

### Other outcomes (RBC and urinary zinc concentrations and, erythrocyte Cu, Zn-SOD)

Results for other zinc outcomes are presented in Table 2. One study reported significant increases in erythrocyte zinc concentration of the exercise group compared to the control group [28]. Another study reported urinary zinc losses, however no statistical significance was reported [30]. Two studies [27,31] reported data on changes in erythrocyte Cu, Zn-SOD activity for three comparisons (Table 2). Significant increase in SOD activity following exercise was reported in the exercise group compared to the control group in one study [27].

**Table 2:** Change in erythrocyte zinc concentration, urinary zinc loss and Cu,Zn SOD activity in the included studies.

Study (author, year)	Outcomes	Change in control group	Change in exercise group	Units	Statistical significance of between group difference
Cordova & Navas 1998	Urinary zinc excretion	-15 ± 208.68	145 ± 173.36	μg/day	NR
Fogelholm 1992	Erythrocyte zinc concentration	-0.01 ± 0.003	0.05 ± 0.004	μmol/g Hb	< 0.001
Azizbeigi *et al*. 2013	Erythrocyte Cu, Zn-SOD	-48.73 ± 232.65	127.53 ± 154.66	U/g Hb	0.014
Lukaski *et al*. 1990 M		-145 ± 379.66	795 ± 387.31	U/g Hb	NR
Lukaski *et al*. 1990 F		-197 ± 347.33	1566 ± 413.22	U/g Hb	NR

Hb, haemoglobin; NR, not reported; SOD, superoxide dismutase

### Risk of bias assessment

**Fig 4:**
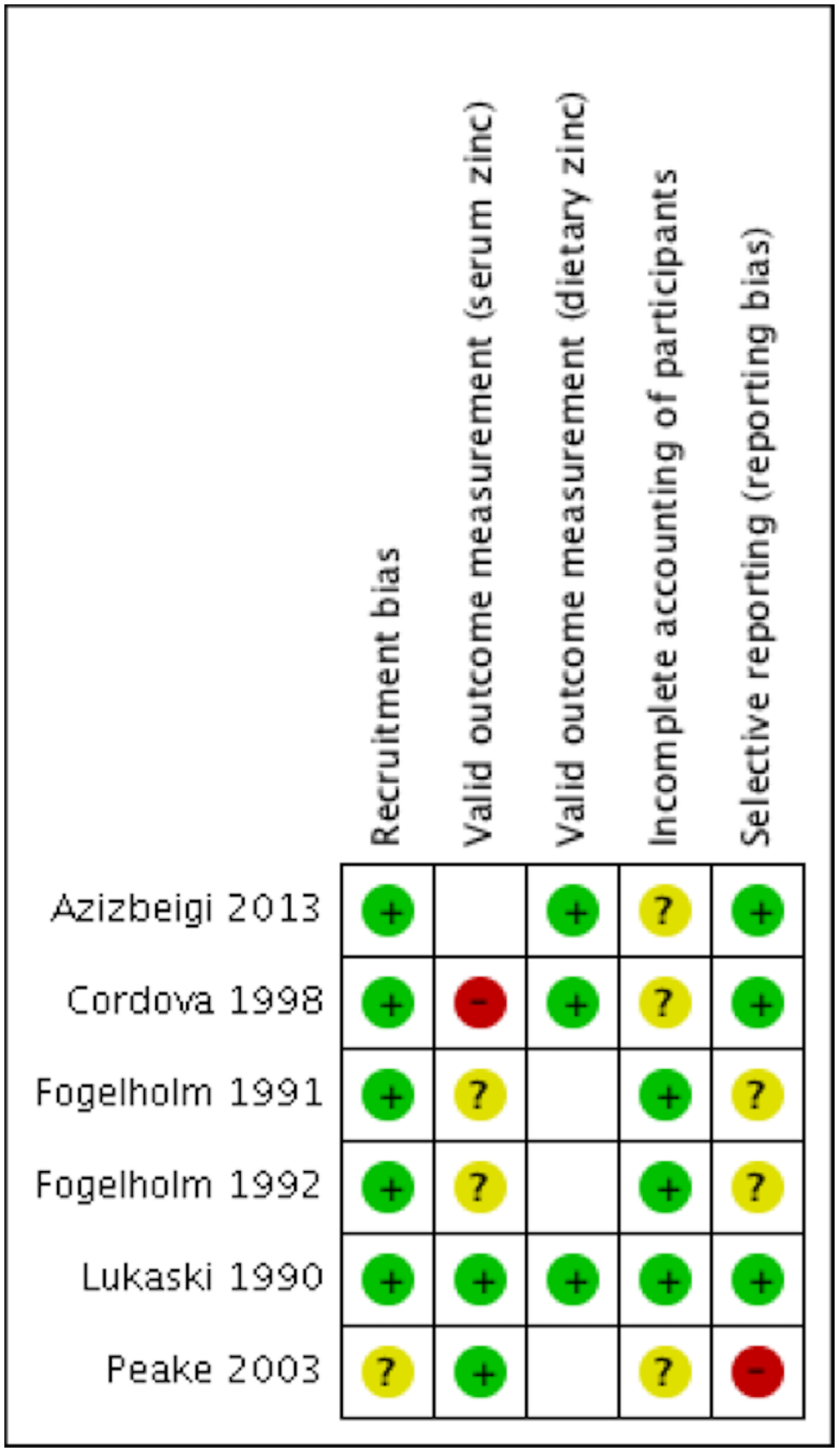
Risk of bias summary judgements on each risk of bias item for each included study. Green (+) symbols represent low risk of bias for the specific criteria for the included study. Yellow (?) symbols represent unclear risk of bias and red (-) symbols denote high risk of bias. Support for judgements is presented in S1 Table.

Fig 4 shows the risk of biases summary for all studies. The support for judgements is presented in S1 Table. The majority of the studies demonstrated *low* risks of bias for recruitment with control group matched in age, sex and BMI with the exercise group [27,28,30–32]. All included studies scored *low* or *unclear* risks of bias for valid measurement of serum zinc, with the exception of one study scoring *high* risk of bias, where non-fasting blood samples were collected for the analysis of serum zinc [30]. Similarly, all of the studies presented with *low* risk of bias for valid measurement of dietary zinc. Most of the studies reported follow up data for all participants thereby scoring *low* risk of bias for incomplete accounting of participants. The majority of the studies reported all relevant outcomes with the exception of one study that failed to report measured dietary intake data [29] and another study which did not present longitudinal changes of dietary zinc intake over the intervention period [28].

## Discussion

The current review of interventional and cohort studies has identified incomplete evidence for the effects of exercise training on zinc status in an apparently healthy adult population. In total, six studies satisfied the inclusion criteria for the present analysis. The limited evidence suggests that exercise is associated with higher dietary zinc intake and erythrocyte SOD activity; while minimal differences were observed in serum zinc concentrations. We have revealed distinct gaps in the current literature regarding the effects of exercise training on zinc status with implications on establishing dietary zinc requirements in populations that exercise regularly.

The studies included in the current review consisted of exercise interventions that are dissimilar in mode, duration and intensity. Three out of six studies included exercise that is predominately aerobic in nature, for example running and swimming [28,29,31]. A mix of aerobic and anaerobic activities were used in two studies [30,32], while another study utilised resistance exercise training only [27]. The heterogeneity in exercise interventions in the included studies contributed to the variance of the outcomes measured. Further, while changes in early markers of exercise training are evident within hours following the first exercise session [33], repeated and progressive exercise bouts are required to develop and maintain beneficial adaptations of exercise. The duration of exercise interventions described in the current analysis ranged from 3 to 24 weeks; therefore, there may be substantial differences in the extent of exercise adaptations presented by the included studies. Combined with the small number of studies in the current review, a quantitative summation of the changes in zinc status, beyond pairwise analyses, was deemed inappropriate.

Based on six comparisons for exercise-induced changes in serum zinc concentration, the estimated mean difference of change for two comparisons indicated that exercise induces significant increase in serum zinc; while one comparison suggested that serum zinc changes are lower in the exercise group compared to control. The current evidence suggests equivocal changes in serum zinc concentration as a result of exercise interventions. The limitations of serum zinc concentration as a marker of zinc status in humans are well described [34]; changes in a multitude of factors, such as inflammation, hormones and age can affect the relationship between zinc status and serum zinc concentration. As such, the majority of studies reported other measures of zinc status, such as erythrocyte and urinary zinc concentration, to describe changes in zinc metabolism under exercise training.

The most commonly measured zinc outcome, other than serum zinc concentration, was erythrocyte Cu, Zn-SOD activity. SOD plays a key role in antioxidative activity by providing catalytic function for the disassociation of the radical oxygen species, superoxide, into oxygen or hydrogen peroxide. In erythrocytes where oxygen exchange occurs with haemoglobin, significant intracellular oxidative stress is induced by exercise acutely through increases in oxygen consumption [35]. As a result, exercise training per se can induce significant increases in SOD activity, with erythrocyte SOD correlating positively to training status [36]. It is unclear from the current evidence whether dietary zinc intake and/or baseline zinc status may influence the exercise-induced increases in erythrocyte SOD activity. Further, while copper status may influence the enzymatic activity of SOD, only two of the included studies reported copper intake of the study populations. Future studies will benefit from the inclusion of copper status to further elucidate the relationship between zinc status and SOD activity. Moreover, the examination of other zinc-dependent enzymes, such as lactate dehydrogenase or carbonic anhydrase, may provide insights into the effects of regular exercise and zinc intake on the enzymatic functions of zinc.

Exercise also impacts on immunity, both acutely and chronically. Following a bout of prolonged high intensity exercise, acute decreases in multiple components of immune function are evident including the reductions in B lymphocyte production of immunoglobulins and antigen-presenting capacity [37]. Whilst the greatest effects of exercise on immune functions are observed following high intensity exercise, prolonged moderate intensity exercise may also exert clinically significant changes in immunity [38]. The acute fluctuations in immune functions contribute to the increased susceptibility to potential pathogens in the hours following exercise. As one of the functions of zinc is to support innate and adaptive immune function, the provision of zinc deficient diets has been associated with lowered immunity in humans [39]. However, the evidence for zinc status in modulating exercise-induced immune changes is less clear. The included study by Peake et al. failed to find significant relationships between plasma zinc levels and immune outcomes following a period of increased training in runners [29]. Further investigations into the relationships between zinc status, immune function and exercise are required.

On the basis of four comparisons, dietary zinc intake appears to be higher in exercise groups compared to control in two comparisons; therefore, we deemed the effects of exercise training on dietary zinc intake to be equivocal. In a study by Lukaski et al., while dietary zinc intake increased in male swimmers over the competition period, zinc density (mg/MJ) remained unchanged [31]. This suggests that higher total zinc intake derived from increased amount of food consumed. In the maintenance of homeostasis, exercise and associated energy expenditure can impact on food and energy intake [40]. It is difficult to elucidate the influence of changing dietary patterns, as a result of exercise, on zinc status and related outcomes. The majority of the included studies did not provide details on the sources of zinc intake or its potential impact on gastrointestinal zinc absorption and zinc status. Further, a number of studies collected dietary data but failed to report quantitative values for dietary zinc intake before and following exercise intervention. The selective reporting of outcomes limited the number of comparisons and evidence available in the present review.

All included studies within this review were low in number of participants and therefore contributed to the limited power of the comparisons in determining the effect of exercise training on markers of zinc status. Further, four out of six included studies recruited men only; similar level of sex bias were noted previously [21], highlighting the sex bias that exists in health research [41]. Moreover, the current review included mostly participants who were highly trained at baseline, for example volleyball players and swimmers. Therefore, the results of this analysis may not be applicable for untrained populations who are initiating exercise training protocols. The implications of the effects on zinc metabolism from initiating exercise training as part of a lifestyle program in patients with chronic diseases, such as diabetes, may be an important consideration particularly in populations at risk of marginal zinc status.

This review is the first to determine the effects of exercise training on markers of zinc status in an adult population. The current report extends our knowledge of the acute effects of exercise on markers of zinc metabolism [20,21]. In the previous reports we described acute fluctuations in serum zinc concentrations in the hours following a bout of aerobic activity, thereby providing the basis to determine the potential long term effects of repeated bouts of exercise on markers of zinc status. One of the strengths of the present review is the determination of risk of biases as per the recommendations by the GRADE working party [24]. In our evaluation, a number of risk of biases were identified, including factors that have implications in the valid measurement of serum zinc concentration, and the risk of selective reporting, particularly in regards to dietary zinc intake. As serum zinc concentrations are affected by numerous factors, including diurnal fluctuations, fasting status and inflammation [1], it is important for investigators to control for potential confounding variables in the determination of the relationship between exercise and serum zinc concentrations. Further, the omission of dietary zinc intake at baseline and following exercise interventions in some studies does not allow for the determination of the effect of exercise on dietary intake and the potential modulation of diet on exercise-induced changes in zinc metabolism. Future studies should consider including additional information regarding zinc density, i.e. amount of dietary zinc compared to energy intake, thereby furthering the understanding of the relationship between energy and zinc intake.

The present review revealed an incomplete evidence base in evaluating the effect of long term exercise training on markers of zinc status. Further, the strength of the presented evidence is limited by the majority of study designs reported being non-randomised or cohort studies. The limited evidence suggests that dietary zinc intake may increase in those who are physically active, by homeostatic adjustments for increases in energy expenditure. Additional studies, with different study populations such as those participating in moderate levels of physical activity, are required to extend the evidence to the general population. Future evaluation on the effects of zinc intake on physical performance will present implications for clinical sports nutrition practice. Exercise training was found to be associated with increases in SOD activity as part of the adaptations of exercise, consistent with the current literature [35]. As the turnover of erythrocyte SOD requires sufficient zinc status to support the adaptations of exercise, the current dietary recommendations for populations who are initiating or continuing exercise intervention should be to consume dietary zinc levels, to at least the Recommended Daily Intake [42].

## Supporting information

S1 Table Risk of bias assessment of included studies. (DOCX)

S1 File PRISMA checklist. (DOC)

S2 File Data extraction. (XLSX)
