# Supplementary material for: Quantifiable effects of regular exercise on zinc status in a healthy population—A systematic review
Source: PLoS One. 2017 Sep 20;12(9):e0184827. doi: 10.1371/journal.pone.0184827 (PMC5607172; doi:10.1371/journal.pone.0184827)
Supplement: S1 Table — (DOCX) [file pone.0184827.s001.docx]

S1 Table. Risk of bias assessment of included studies

Azizbegi et al. 2013

| Item | Authors’ judgement | Support for judgement |
| --- | --- | --- |
| Recruitment | Low | Appropriate control group with matching age, sex and BMI |
| Valid outcome measurement (serum zinc) | N/A |  |
| Valid outcome measurement (dietary zinc) | Low | Appropriate methods of assessment with macronutrients reported. Unclear on supplement use |
| Incomplete accounting of participants | Unclear | No report of complete rates |
| Selective outcome reporting | Low | All relevant outcomes reported |

Cordova & Navas 1998

| Item | Authors’ judgement | Support for judgement |
| --- | --- | --- |
| Recruitment | Low | Control group with matching age, sex and BMI. Control group described as moderately trained (7h/week of exercise) |
| Valid outcome measurement (serum zinc) | High | Non-fasting blood sampling for serum zinc |
| Valid outcome measurement (dietary zinc) | Low | Appropriate method of assessment with energy reported. Unclear on supplement use |
| Incomplete accounting of participants | Unclear | No report of completion rates |
| Selective outcome reporting | Low | All relevant outcomes reported |

Fogelholm et al. 1991

| Item | Authors’ judgement | Support for judgement |
| --- | --- | --- |
| Recruitment | Low | Matched control group for age, sex and BMI |
| Valid outcome measurement (serum zinc) | Unclear | Unclear on whether investigators used trace element free tubes for blood collection |
| Valid outcome measurement (dietary zinc) | N/A |  |
| Incomplete accounting of participants | Low | Table of results matched initial recruitment numbers |
| Selective outcome reporting | Unclear | Did not report absolute dietary zinc intake (mg/d) or values for before or after exercise intervention |

Fogelholm 1992

| Item | Authors’ judgement | Support for judgement |
| --- | --- | --- |
| Recruitment | Low | Control group matched for age, sex and BMI |
| Valid outcome measurement (serum zinc) | Unclear | Unclear on whether investigators used trace element free tubes for blood collection |
| Valid outcome measurement (dietary zinc) | N/A |  |
| Incomplete accounting of participants | Low | All participants accounted for |
| Selective outcome reporting | Unclear | Did not present dietary zinc intake data before and after intervention |

Lukaski et al. 1990

| Item | Authors’ judgement | Support for judgement |
| --- | --- | --- |
| Recruitment | Low | Control group matched for age, sex and body composition |
| Valid outcome measurement (serum zinc) | Low | Appropriate collection and analytical methods |
| Valid outcome measurement (dietary zinc) | Low | Appropriate method and reporting of energy and supplements |
| Incomplete accounting of participants | Low | All participants completed |
| Selective outcome reporting | Low | All relevant outcomes reported |

Peake et al. 2003

| Item | Authors’ judgement | Support for judgement |
| --- | --- | --- |
| Recruitment | Unclear | Control group (BMI > 25 kg/m^2^) different in BMI to exercise group |
| Valid outcome measurement (serum zinc) | Low | Appropriate collection and analytical methods |
| Valid outcome measurement (dietary zinc) | N/A |  |
| Incomplete accounting of participants | Unclear | No report of completion rates |
| Selective outcome reporting | High | Collected dietary intake data but did not report quantitatively |
